# The role of endoscopy in gastric button battery ingestions: A case and literature review

**DOI:** 10.1002/jpr3.12116

**Published:** 2024-08-05

**Authors:** Mark Ambert, Jasmine Patterson, Racha Khalaf

**Affiliations:** ^1^ Department of Anesthesiology and Perioperative Medicine University of South Florida Morsani College of Medicine Tampa Florida USA; ^2^ Department of Internal Medicine, Division of Emergency Medicine University of South Florida Morsani College of Medicine Tampa Florida USA; ^3^ Department of Pediatric Gastroenterology University of South Florida Morsani College of Medicine Tampa Florida USA

**Keywords:** corrosive ingestion, foreign body ingestion, magnet

Currently, the precise timing and management of gastric button battery removal remains controversial. Generally, the algorithms differentiate treatment based on age, length of battery retention, and symptoms.

The European Society of Paediatric Gastroenterology and Nutrition (ESPGHAN) recommends follow‐up imaging 7–14 days after ingestion to reduce unnecessary endoscopic exposure.^1^ Direction provided from The National Capital Poison Center guideline states that gastric button batteries should be immediately removed only if symptomatic (emesis, bleeding) and if asymptomatic, patients should be followed up with imaging.^2^ The Children's Hospital of Colorado's policy is in accordance with 2015 guidelines from The North American Society of Pediatric Gastroenterology and Nutrition (NASPGHAN), which allow for the discretion of the physician to determine management of gastric button batteries.^3^ Factors such as button batteries greater than 20 mm and patient age under 5 years support the decision to endoscopically remove, per the NASPGHAN endoscopic committee.^3^

Herein, we describe the case of a 23‐month‐old female who presented asymptomatically to the emergency department 4 h after ingesting an 11.6 × 5.4 mm button battery. The emergency room performed a computed tomography (CT) scan to confirm the location of battery, then consulted the pediatric gastroenterology for evaluation. Based on the patient's age, we elected to remove the button battery 9 h after ingestion. At the time of endoscopy, there was significant superficial erosion of the gastric mucosa and as we attempted removal, the negative pole of the battery was actively creating ongoing injury of the mucosa. Our experience was consistent with some prior studies, in observing gastric mucosal injury in a patient who presented asymptomatically.^4^ A previous multicenter review showed that among 68 patients who underwent endoscopic gastric button battery removal, 60% had evidence of gastric mucosal injury despite only 25% being symptomatic.^4^ The risk for gastric mucosal injury was statistically significant and correlated significantly with duration of retention (*p* = 0.0018).^4^

This case showcases the possibility for button battery injury to the gastric mucosa even in asymptomatic patients following ingestion. Early endoscopic removal can be considered as a management option in addition to observation alone. Larger, prospective studies are needed to better understand the relationship (Figure 1).

**Figure 1:**
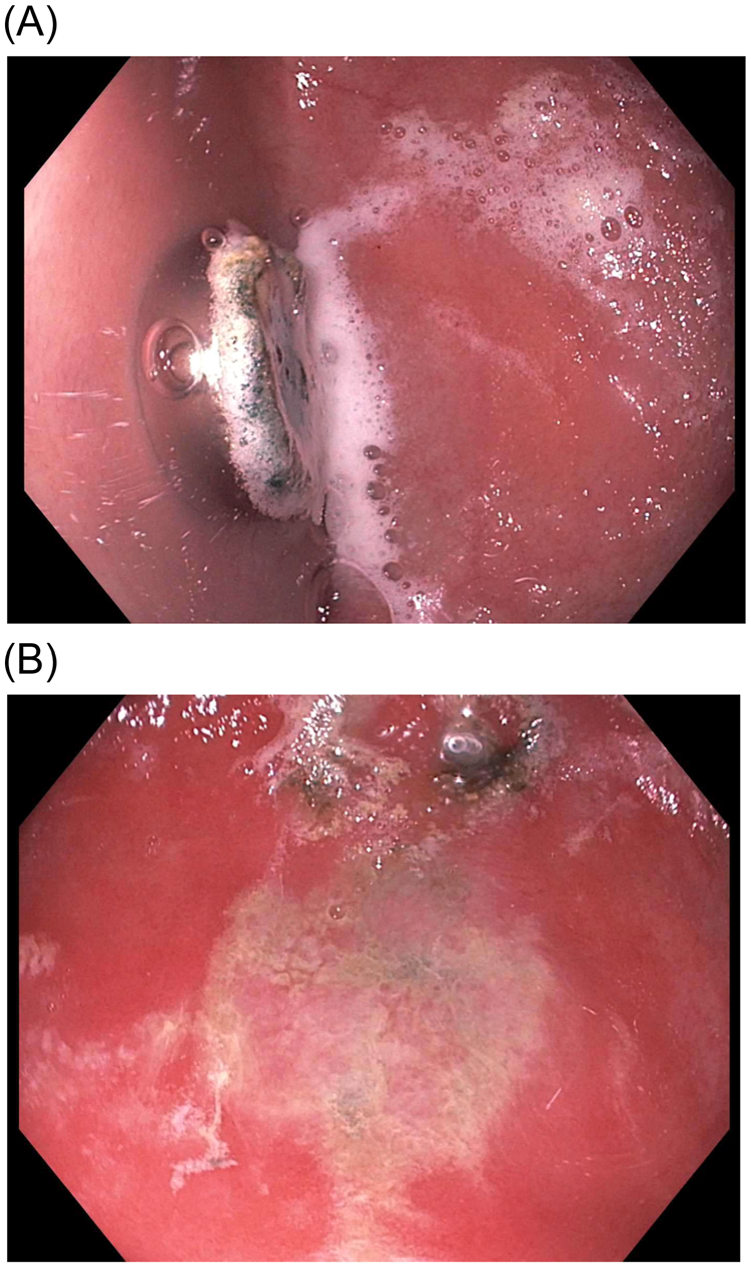
(A) Endoscopic photograph displaying 11.6 × 5.4 mm button battery in the gastric body; active erosion is occurring at the negative pole of the battery. (B) Endoscopic photograph demonstrating a large mucosal erosion present on the patient's gastric body.

## CONFLICT OF INTEREST STATEMENT

The authors declare no conflict of interest.

## ETHICS STATEMENT

The patient's mother provided verbal consent for the use of the patient's case.
